# A case of an Angelman-syndrome caused by an intragenic duplication of *UBE3A* uncovered by adaptive nanopore sequencing

**DOI:** 10.1186/s13148-024-01711-0

**Published:** 2024-08-02

**Authors:** Laura Holthöfer, Stefan Diederich, Verena Haug, Lioba Lehmann, Charlotte Hewel, Norbert W. Paul, Susann Schweiger, Susanne Gerber, Matthias Linke

**Affiliations:** 1 Institute for Human Genetics, University Medical Center of the Johannes Gutenberg University Mainz, Mainz, Germany; 2 Neuropediatrics, University Medical Center of the Johannes Gutenberg University Mainz, Mainz, Germany; 3 Institute for History, Philosophy, and Ethics of Medicine, Johannes Gutenberg-University Medical Center Mainz, Mainz, Germany

## Abstract

Adaptive nanopore sequencing as a diagnostic method for imprinting disorders and episignature analysis revealed an intragenic duplication of Exon 6 and 7 in *UBE3A* (NM_000462.5) in a patient with relatively mild Angelman-like syndrome. In an all-in-one nanopore sequencing analysis DNA hypomethylation of the *SNURF*:TSS-DMR, known contributing deletions on the maternal allele and point mutations in *UBE3A* could be ruled out as disease drivers. In contrast, breakpoints and orientation of the tandem duplication could clearly be defined. Segregation analysis in the family showed that the duplication derived de novo in the maternal grandfather. Our study shows the benefits of an all-in-one nanopore sequencing approach for the diagnostics of Angelman syndrome and other imprinting disorders.

**Supplementary Information:**

The online version contains supplementary material available at 10.1186/s13148-024-01711-0.

## Introduction

Angelman syndrome (AS, OMIM #105,830) patients were first described by Angelman [1] and are clinically characterized by severe intellectual disability and speech impairment as well as ataxia, epilepsy and distinct behavioral profiles [21]. It is caused by a loss of function of the maternal copy of the gene encoding the ubiquitin ligase E3A (*UBE3A*, OMIM * 601,623) and other genes on chromosome 15q11–13 [21]. Known as a genomic imprinting disorder, AS is caused by genetic or epigenetic defects, that lead to the disruption of imprinted genes. Human genomic imprinting in turn is defined by epigenetic modifications of a small set of human genes based on their parental origin, that lead to their monoallelic expression [24].  AS is caused by four possible molecular pathomechanisms: (1) interstitial deletions of 5–7 Mb spanning the imprinted region on 15q11.2q13 on the maternal allele (70–75% of AS cases), (2) maternally inherited *UBE3A* point mutations (5–10%), (3) imprinting defects causing aberrant DNA methylation within chromosome 15q11–q13 that disrupt the expression of maternally inherited *UBE3A* (3–5%) and (4) uniparental disomy (UPD) of the paternal chromosome 15q11–q13 (2–3%) [5, 7]. Maternally derived duplications affecting several Mb in the 15q11-q13 region and their genotype–phenotype correlation have been previously reported [2]. However, they seem to cause entities distinctive from AS. The smallest duplication in the 15q11-q13 region as part of a detailed case report, encompassed the complete *UBE3A* gene and was found in patients with developmental delay and neuropsychiatric symptoms [14]. The ClinVar-Database contains seven entries of partial heterozygous *UBE3A* duplications (> 50 bp, Supp. Table 1), ranging from 59 bp to 70 kb size.

Technical and bioinformatical developments over the last 12 months have led to a significant improvement in sequencing accuracy of Oxford Nanopore Technologies (ONT) sequencing platforms thereby providing a long-read sequencing tool which, together with its capability for copy number change and DNA methylation detection as well as phasing of parental alleles for homozygosity analysis, can cover virtually all putative molecular mechanisms of AS in an all-in-one approach [22]. One exception with regard to AS is thought to be the discrimination between an epimutation at the *SNURF*:TSS-DMR and a paternal heterodisomy without genotyping closely related family members [9]. Theoretically, adaptive sampled (or WGS) Nanopore sequencing methylation data on several other DMRs on the same chromosome could be used to solve the majority of such cases. In view of the above, Nanopore sequencing represents a versatile first-tier diagnostic tool for imprinting disorders and episignature analysis replacing several conventional techniques such as (MS)-MLPA, Bisulfite Pyro- and DNA Sanger Sequencing that were part of a traditional stepwise diagnostic process. Furthermore, the technology offers the unique possibility to bypass wet lab enrichments by a bioinformatic approach that uses the possibility of reversing the voltage across pores to enable selection of fragments for sequencing based on real-time assessment of a small initial part of a read. Known as adaptive sampling [12], this feature can be used to enrich regions of interest or deplete unwanted fragments.

Here we report on a family with an intragenic tandem duplication of Exon 6 and 7 in *UBE3A*. The index patient was diagnosed with AS-like syndrome. The maternally inherited copy number change was uncovered by adaptive nanopore sequencing, with which duplication breakpoints and orientation of the duplication could be clearly defined. In line with the expected segregation of Angelman syndrome in the family, the de novo duplication in the unaffected mother of the index case was located on the allele inherited from her father.

## Material and methods

### Case presentation

Our patient is the first and only child of nonconsanguinous parents. The pregnancy was the result of IVF (couple sterility of unexplained cause) and was completely unremarkable (no gestational diabetes, no medication, and no indication of infections or fetal growth disorders). Due to a pathological CTG and a labor arrest cesarean section at 39 + 2 gestational week (GW) was carried out. While, birth weight (2960 g, 10th percentile) and length (51 cm, 32nd percentile) were normal, microcephaly was diagnosed (head circumference 32 cm, below 1st percentile,  − 2.6 z).

Early on, the parents had the impression that their son was not developing appropriately. A first presentation in the neuropediatric outpatient clinic took place at the age of 14 months, when a clear developmental delay (no free sitting, no crawling) with dystrophy and microcephaly was diagnosed. EEG examination revealed pathological findings in terms of beta disturbance and epileptic potentials. At the age of 17 months EEG was found unchanged. No seizures were reported by the parents. MRI examination of the brain revealed unremarkable findings, as did echocardiography, ultrasonography of the abdomen, X-ray examination of the thorax, and basic metabolic screening. Endocrinologic causes for the dystrophy were excluded.

Initial presentation to our genetic counseling center was at 19 months of age. The patient presented with a friendly demeanor, delayed developmental milestones (no sitting, no walking without support, and no words) and reduced (< 1st percentile) weight (8500 g, − 2.4 z), height (77.1 cm, − 2.1 z), and head circumference (43.9 cm, − 4.1 z). He showed subtle dysmorphic features such as large mouth, small chin, prominent nose and mildly deep set ears.

EEG at 33 months of age was unremarkable. Due to pronounced sleep disturbances, melatonin was administered in phases.

At 34 months of age, our patient developed Kawasaki syndrome with coronary ectasia.

At the time of the last clinical reevaluation, the patient was 40 months old. Head circumference (46 cm, − 3.88 z), length (88 cm, − 2.7 z), and weight (11 kg, − 2.6 z) were below the first percentile. Seizures had not occurred. He could walk a few steps but was very insecure and he still explored a lot with his mouth. Salivation was increased. He was vocalizing but did not speak. He has a very friendly disposition and laughs a lot.

A typical but comparatively mild course of Angelman syndrome with dystrophy, short stature, microcephaly and global developmental delay with severe speech delay was diagnosed. EEG examinations initially showed abnormal findings, the last EEG had been inconspicuous and seizures had not occurred.

The family history was unremarkable.

### Ethical dimension

This clinical case is reported under the premises of broad consent, that is the consent of patients and/or their legal proxies that clinical data and biological samples collected in the course of diagnostics and treatment are used for research purposes. This concept of broad consent has been acknowledge as a standard procedure for the Johannes Gutenberg University Medical Center by the regulatory authority, the Ethics Commission of the Chamber of Physicians in Rhineland-Palatinate. This regulatory board, however, is responsible for the ethical approval of clinical studies with sufficient numbers of participants to generate statistical power. Healing attempts in single cases like off-label use of cancer medication e.g. for childhood cancer or compassionate release of beneficial study drugs for single patients outside a study are controlled by the clinical ethics committee of the Johannes Gutenberg University Medical Center. In the specific case reported here, we informed the family about the use of diagnostic data and subsequent findings in biological material collected during diagnostics and treatment in the setting of case reports and publications. The clinical ethics committee agreed to include the following statement in our paper: This case report is a relevant contribution to clinically relevant research. Informed consent was granted by broad and individual consent. This includes information about the fact that both, the nature of research and the nature of the clinical case may lead to a situation in which a willing and technically able third party may be able to relate data and reported findings to an individual person. Consent was given under these premises and thus the clinical ethics committee of the Johannes Gutenberg University Medical Center in Mainz, Germany, has (a) no ethical concerns regarding the study in principle; (b) concludes that the ethical questions does not need to be addressed in the framework of clinical studies and thus by the Ethics Commission of the Chamber of Physicians based on the fact, that a single case is reported; (c) weighs the informed consent and expressed autonomy of the patient and/or parents or legal proxies against the risk of relating data and findings to an individual person and thus comes to the conclusion that the underlying research is ethically sound and the presented research including data and findings can justifiably be published.

### DNA isolation

Genomic DNA from peripheral blood was extracted by Gentra Puregene Blood Kit (Qiagen, Hilden, Germany) according to the manufacturer’s instructions, followed by quality (NanoDrop) and quantity (Qubit) assessment. Extracted DNA was stored at − 20 °C until further use.

### Nanopore sequencing and bioinformatic processing

Native barcoding sequencing libraries were prepared from 400 ng genomic DNA using the Native Barcoding Sequencing kit SQK-NBD114.24 (ONT, Oxford Nanopore Technologies Ltd., Oxford, UK) following the manufacturer’s protocol. The clean-up step after adapter ligation was intended to size-select fragments and was done with Long Fragment Buffer. The barcoded patient library, among others, was loaded on a single R10.4.1 (FLO-PRO114M) flow cell and sequenced on a PromethION 24 device within 72 h. MinKNOW (v23.06.04) was used to supervise the initial sequencing run, including adaptive sampling with enrichment of intended genomic regions by setting human genome build hg19 as input reference. Genomic regions subject to medically relevant parent-of-origin methylation or Epi-variants as potential contributors to hereditary conditions [3, 8, and 16] were set as the target regions in the BED format file. The entire genomic and 5 kb flanking sequence of genes associated with the criteria above was also subjected to adaptive sampling. The total size of the target regions was 24,431,679 bps. Information on target regions will be available upon request. Base-calling and alignment to the human reference genome (hg19) via Minimap2 [11] was performed using Dorado software from ONT (v0.3.4) with a super accuracy model with base modifications (dna_r10.4.1_e8.2_400bps_sup@v4.2.0_5mCG_5hmCG@v2). Haplotype-aware small variant calling was accomplished using DeepVariant (v1.5, model “ONT_R104”) [15]. Phasing of reads was performed with whatsapp (v2.0) which uses nanopore long reads to link adjacent single nucleotide variants and then phases the mapped reads to infer the haplotypes [13]. The structural variations were called using Sniffles (version 2.0.3) [23]. Copy numbers were analyzed from the aligned files utilizing the CNVpytor software (v1.3.1), which discovers and analyses copy number variations and alterations based on read depth [17].

### MLPA

100 ng of DNA were used together with the SALSA MLPA Probemix P336 UBE3A-B1 (MRC Holland, Amsterdam, NL) and used for DNA copy number quantification according to the manufacturer’s instructions. Copy number analysis was performed using GeneMarker software v.3.0.1.

### Pyrosequencing

500 ng of DNA were bisulfite treated by the EZ DNA Methylation-Direct Kit (Zymo, Irvine, USA). 100 ng of bisulfite treated DNA were PCR amplified (Table 1) by FastStart Taq DNA Polymerase (Roche, Basel, CH) according to the manufacturer’s instructions. Afterwards quantification of DNA methylation was carried out by Pyrosequencing (Qiagen, Hilden, GER) which offers better resolution than MS-MLPA.

**Table 1:** PCR-Primer

Pyrosequencing				
Gene (RefSeq ID)		5-3’ Primer sequence	Product length	References
*SNURF*:TSS-DMR	For	Bio-AGGGAGTTGGGATTTTTGTATT	237 bp	White et al. [20]
	Rev	CCCAAACTATCTCTTAAAAAAAAC		
	Seq	ACACAACTAACCTTACCC (3 CpGs)		
rs_77329250	For	AACCCATTTAAAATGAAATCAAAG	103 bp	
	Rev	Bio-GTCCGGCCTATGTTGTTTAATTT		
	Seq	AAAGAGTAAAAAATACTTAG		
Sanger sequencing				
*UBE3A* Breakpoint	For	GGGTGGATCACATGGTCAGG	438 bp	
	Rev	TGACCGAACAATTGATGGAGGT		

Segregation analysis to elucidate the parental origin of the copy number change that occurred de novo in the unaffected mother was performed by genotype analysis. Therefore, 100 ng of genomic DNA from the mother and her parents were PCR amplified (Table 1) by FastStart Taq DNA Polymerase according to the manufacturer’s instructions. Afterwards absolute quantification of allele frequency was carried out by Pyrosequencing (v.2.5.10).

### Sanger sequencing of duplication breakpoint

100 ng of DNA were PCR amplified (Table 1) and sequenced on a SeqStudio Flex Genetic Analyzer (ThermoFisher Scientific, Waltham, USA) according to the manufacturer’s instructions. Sequence analysis was performed using Mutation Surveyor software v.5.1.

## Results

### Copy number analysis

**Fig.1:**
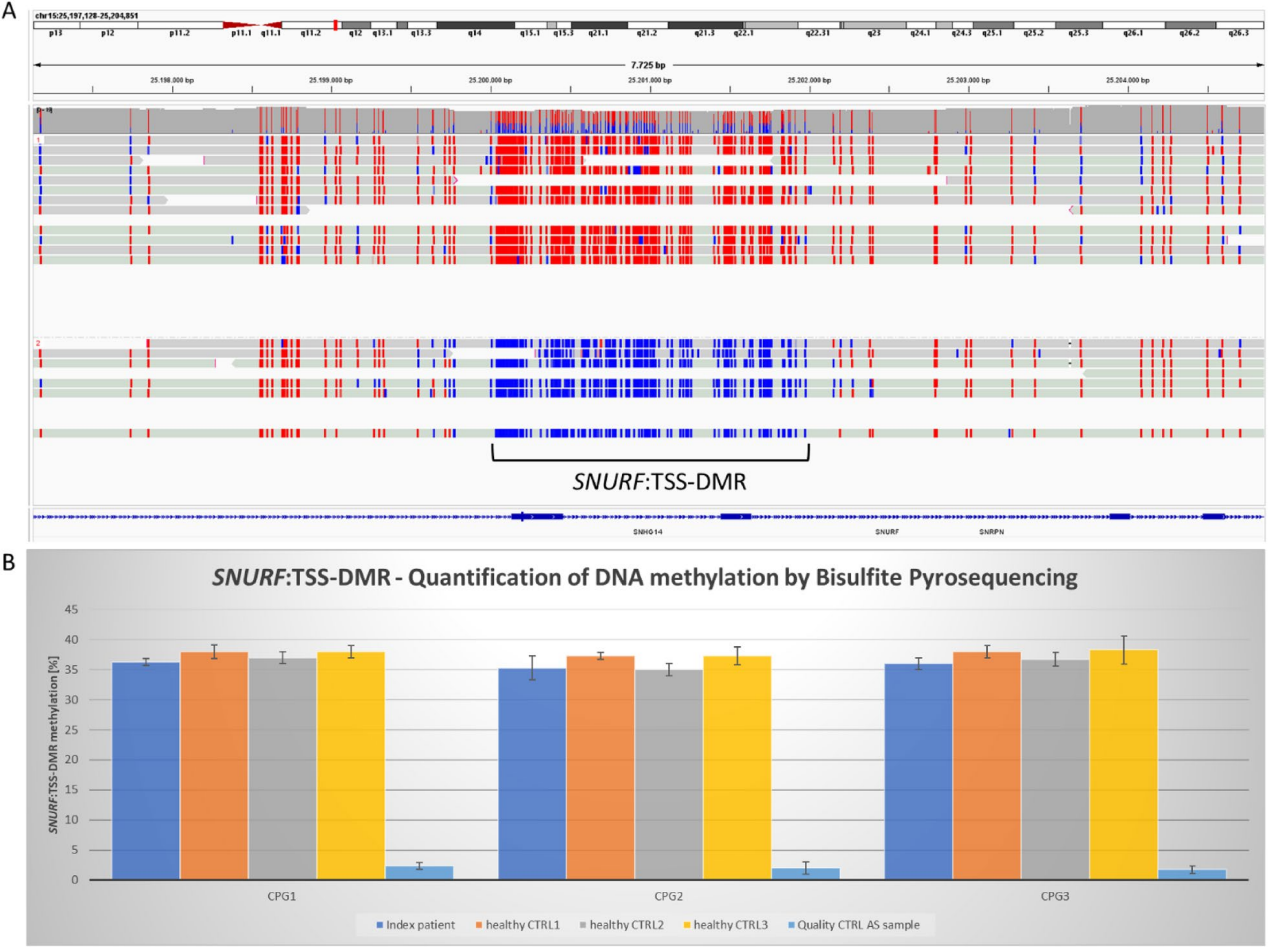
**A** Integrative genomics viewer (IGV) screen capture images of bam files showing the *SNURF*:TSS-DMR (primary DMR). Both haplotypes and their corresponding DNA methylation are shown (top: maternal haplotype with methylated CpGs in red, bottom: paternal haplotype with unmethylated CpGs in blue) **B** Quantification of DNA methylation of the *SNURF*:TSS-DMR by Bisulfite Pyrosequencing of 3 CpGs. Mean methylation plus standard deviation of three technical replicates per sample are shown. From left for each CpG: index patient, healthy controls 1-3 and a sample of a known Angelman syndrome patient (maternal class I deletion on Chr.15q11.2q13) serving as an assay quality control

A large interstitial deletion of 5–7 Mb in the imprinted region 15q11.2q13 on the maternal allele was ruled out based on DNA methylation quantification of the *SNURF*:TSS-DMR by nanopore and pyrosequencing (Fig. 1A) as well as copy number analysis of chromosome 15q11.2q13 using nanopore data (Supp. Figure 2).

**Fig. 2:**
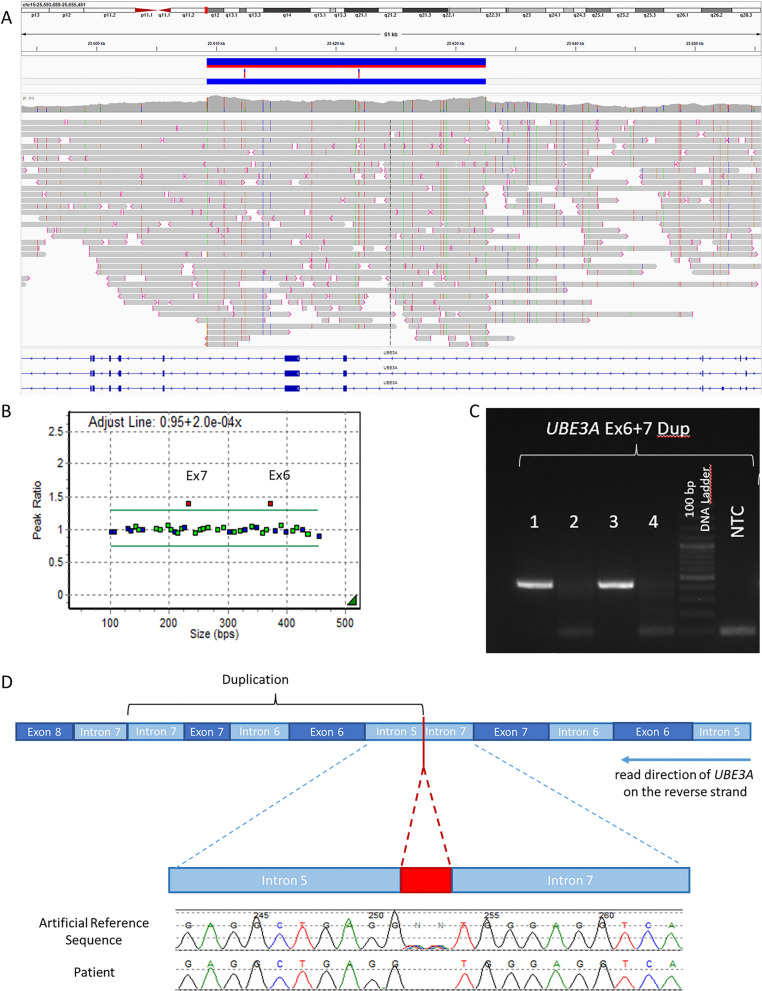
**A** IGV screen capture images showing breakpoints of the *UBE3A* duplication of Exon 6 and 7. **B** Peak Ratio Plot MLPA **C** Breakpoint-PCR between Intron 7 and Intron 5. Sample 1: mother of the index patient, 2: father of the index patient, 3: index patient, 4: healthy proband, NTC: no template control. **D** Sanger Sequencing of the Breakpoint-PCR product. Artificial Reference Sequence: bioinformatically combined sequence of intron 5 and intron 7 at the breakpoint detected by Nanopore Sequencing. Inserting two “NN‘s” at the breakpoint for better breakpoint visibility

Instead, an intragenic heterozygous duplication of Exon 6 and 7 (NM_000462.5) was identified by nanopore sequencing and confirmed by MLPA copy number analysis (Fig. 2A and B). Size (23,340 b), localization and orientation of the tandem duplication in *UBE3A* was resolved by nanopore sequencing (Fig. 2A). MLPA analysis confirmed the duplication in the mother of the index patient but not her parents, indicating a de novo event on one of her parental alleles. The duplication breakpoints could be validated by breakpoint-PCR (Fig. 2C) plus subsequent Sanger Sequencing (Fig. 2D) and resulted in the following karyotype of the index case (ISCN 2020): seq[GRCh38] NC_000015.10:g. 25364087-25387427dup mat.

The copy number change is predicted to be an out-of-frame duplication by the Reading Frame Checker of the Leiden Open Variation Database (LOVD v.3.0) on *UBE3A* transcript NM_000462.3.

### Analysis of *UBE3A* genomic sequence

Nanopore sequencing revealed no SNVs in *UBE3A* that would lead to the observed phenotype of the index patient. Sequencing data of *UBE3A* showed 100% horizontal coverage of the entire gene with a mean vertical coverage of > 10 x.

### Quantification of DNA methylation

Phased nanopore reads revealed no aberrant DNA methylation signature in the *SNURF*:TSS-DMR of the index patient (Fig. 1A). Quantification of DNA methylation in the *SNURF*:TSS-DMR by Bisulfite Pyrosequencing showed no differences compared to three unaffected control samples (Fig. 1B).

Additional to the *SNURF*:TSS-DMR, other DMRs on Chromosome 15 were analyzed in detail based on methylation status calculated from nanopore data. Quantification of DNA methylation in our index patient revealed no aberrant patterns in the *NDN*:TSS-DMR (*Locus Reference Genomic identifier*: LRG_1047), *IGF1R*:Int2-DMR (LRG_1055) and *MKRN3*:upstream enhancer region (LRG_1045) (Supp Fig. 1).

### Segregation analysis

**Fig. 3:**
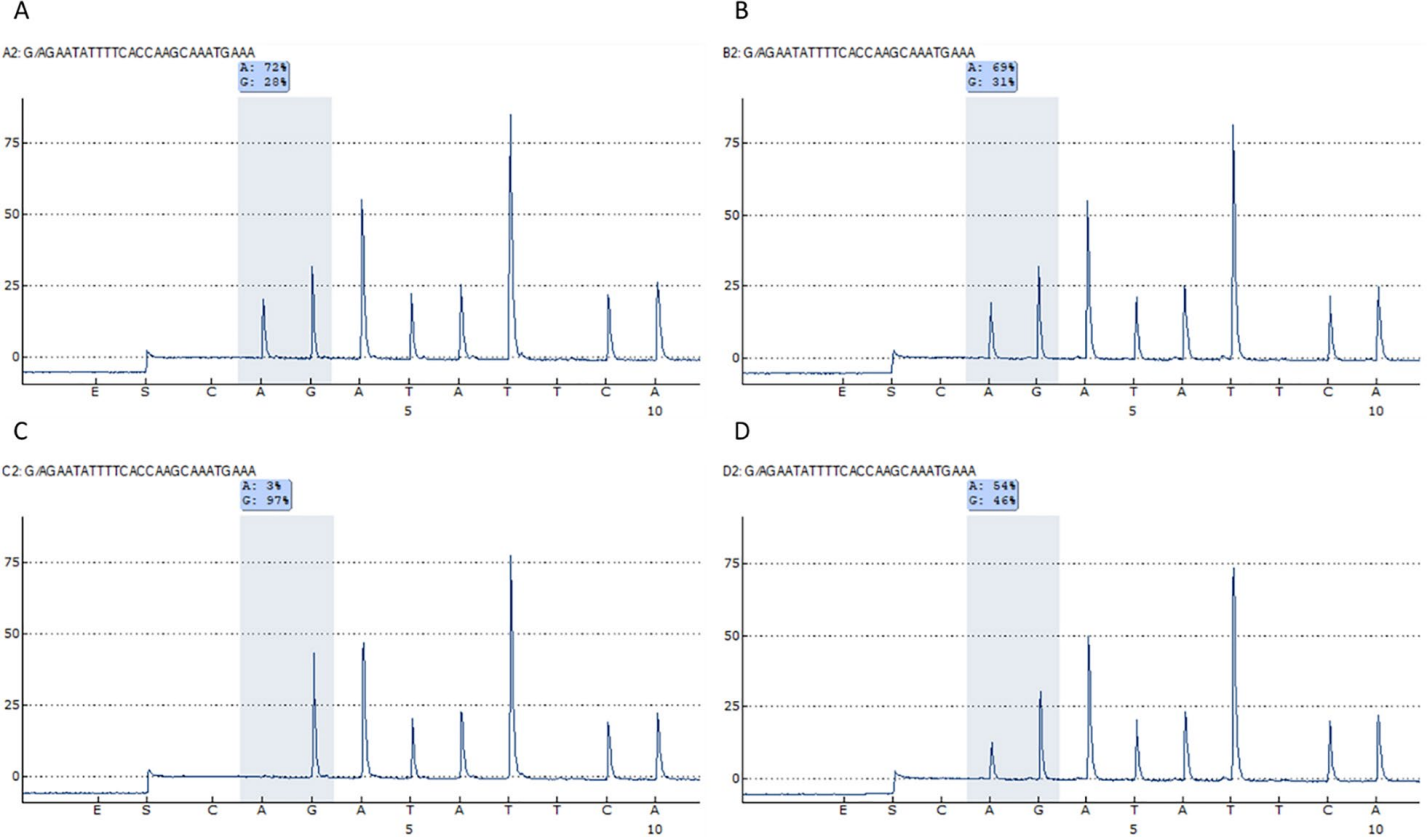
Segregation analysis by Pyrosequencing of rs77329250. **A** index case, B mother of index case, **C** maternal grandmother of the index case, **D** maternal grandfather of the index case

Based on nanopore sequencing data of the index case, allele frequency determination of several heterozygous SNPs throughout the duplicated region pointed towards a skewed ratio and thus potentially allow for further segregation analysis throughout the family. Pyrosequencing of rs77329250 that is located in the duplicated region of *UBE3A* confirmed ^1^/_3_:^2^/_3_ ratios of G:A in the index case and his mother, indicating that Adenine is associated with the heterozygous duplication (Fig. 3). The maternal grandmother showed homozygosity for Guanine at rs77329250, thus Adenine must be inherited by the maternal grandfather. This constellation is in line with a de novo event on the paternal inherited allele in the unaffected mother of the index case.

## Discussion

To our knowledge, this is the first case report on a family with an intragenic tandem duplication of Exon 6 and 7 in *UBE3A* causing Angelman syndrome. The maternally inherited copy number change was uncovered by adaptive nanopore sequencing. Consistent with the inheritance pattern in Angelman syndrome, we have been able to detect the de novo duplication on the paternally inherited allele in the unaffected mother of the index case.

The detailed characterization of the copy number change (breakpoints and orientation) was done using nanopore sequencing data showing that the heterozygous duplication was placed in a tandem and non-inverted orientation within the *UBE3A* gene. Previous studies on duplications in 15q11-q13 [14] used chromosomal microarray analysis (CMA) and clinical testing often involves (MS)-MLPA to analyze copy number changes [6], thus making an unambiguous statement about the location and orientation of a duplication impossible. Based on the provided karyotype, accessible ClinVar entries with regard to partial duplications of *UBE3A* also suggest that CMA or Exome sequencing was performed. Nanopore sequencing assisted phasing of parental alleles allows for detection of uniparental isodisomies by analyzing loss of heterozygosity, thus allows for the discrimination between this type of UPD and an epimutation at the *SNURF*:TSS-DMR as the molecular cause of AS. Heterodisomies and epimutations at imprinted loci are difficult to distinguish if parental DNA is not available for segregation analysis [9]. However, adaptive nanopore sequencing could overcome this problem by the enrichment of further primary or secondary DMRs on the same chromosome. Assuming that the chosen regions underlie genomic imprinting in the starting material to be analyzed and are not known for multilocus imprinting disturbances (MLID) [10], they should show unaffected methylation patterns if an epimutation is the cause of an imprinting syndrome, while a complete heterodisomy of the affected chromosome is expected to show aberrant methylation patterns of the additional DMRs as well. As anticipated in a case of AS caused by an intragenic *UBE3A* copy number change, DNA methylation quantification revealed no aberrant patterns in the *NDN*:TSS-DMR, *IGF1R*:Int2-DMR as well as in the *MKRN3*:upstream enhancer region on Chromosome 15. Further studies on patients affected by imprinting disorders that are known to be caused by uniparental heterodisomies could prove our proposed scenario.

Since nanopore long-read sequencing technology can also detect DNA modifications without any additional wet lab effort, nanopore sequencing is a first-tier diagnostic method for elucidating all of the molecular causes of AS and other imprinting disorders in an all-in-one approach. Adaptive sampling is a unique feature of this sequencing technology that does not need wet lab-intensive enrichment of target regions and allows for multiplexing samples with sufficient vertical coverage on the same flowcell [12]. Currently the application still has the drawback that relatively large target regions have to be selected in order for the whole process to run effectively. Currently, further developments in bioinformatics are addressing this problem [18, 19] and significant efficiency improvements can be expected in the near future as a result.

### Supplementary Information

Additional file 1.

## Data Availability

The data that support the findings of this study are not openly available due to reasons of sensitivity and to preserve individuals’ privacy under the European General Data Protection Regulation, but are available from the corresponding author upon reasonable request.
