# Supplementary material for: A case of an Angelman-syndrome caused by an intragenic duplication of UBE3A uncovered by adaptive nanopore sequencing
Source: Clin Epigenetics. 2024 Aug 2;16:101. doi: 10.1186/s13148-024-01711-0 (PMC11297752; doi:10.1186/s13148-024-01711-0)
Supplement: Supplementary file 1 — Additional file 1. [file 13148_2024_1711_MOESM1_ESM.docx]

**
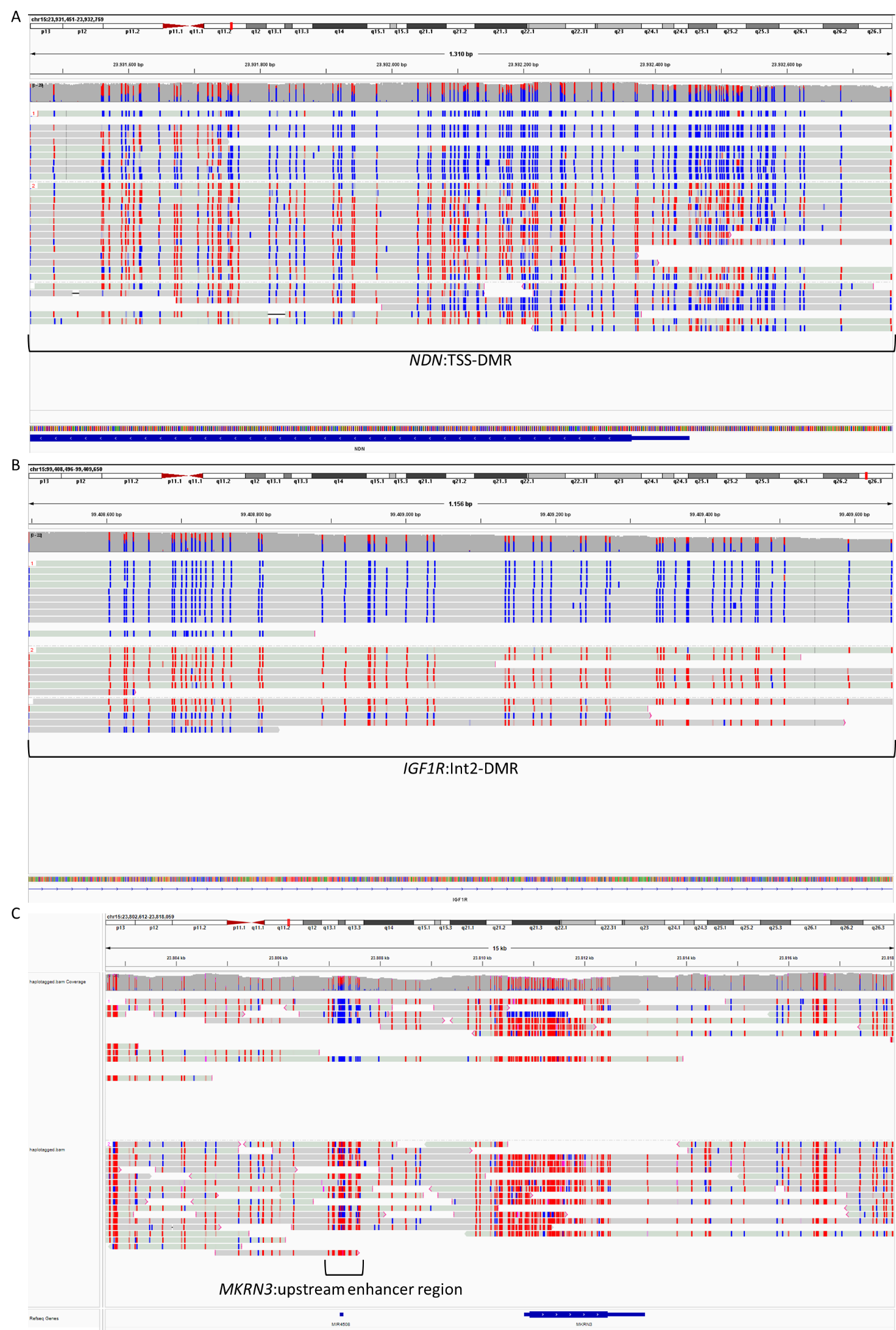
Supp. Fig.1:** DNA methylation quantification of three more DMRs on Chromosome 15 based on Nanopore sequencing data of the index patient. A: *NDN*:TSS-DMR (secondary DMR), B: *IGF1R*:Int2-DMR (primary DMR) and C: *MKRN3*:upstream enhancer region (Back 2020, secondary DMR).


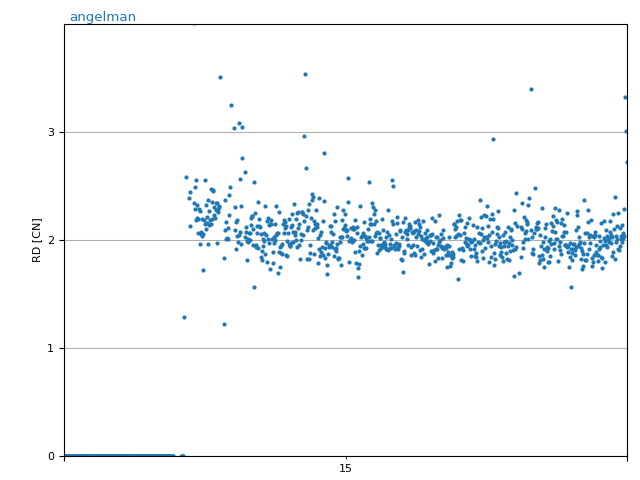


**Chr.15q11.2-q13**

**Supp. Fig.2:** Copy number analysis of chromosome 15. Manhattan plot was generated by CNVpytor with bin size of 100000. Vertical axis represents the relative copy number in log2 ratio, horizontal axis shows relative genome coordinate of chromosome 15 from the centromere to telomere of the long arm (from left to right).

**Supp. Table 1:** Summary of ClinVar entries with partial *UBE3A* duplications and completed by the present case

| ClinVar entry | Genomic location (GRCh38) | Annotation | Size | Pathogenicity |
| --- | --- | --- | --- | --- |
| VCV000155977.1 | chr15:  25339174-25339175 | NC_000015.10:g.25339175_  25339233dup | 59 bp | Pathogenic |
| VCV000144752.2 | chr15: ?_25337273-25342717_? | NC_000015.10:g.(?_25337273)_  (25342717_?)dup | 5,445 bp | VUS |
| VCV000148472.2 | chr15: ?_25365789-25375524_? | NC_000015.10:g.(?_25365789)_  (25375524_?)dup | 9,736 bp | VUS |
| VCV000149842.2 | chr15: ?_25334870-25351819_? | NC_000015.10:g.(?_25334870)_  (25351819_?)dup | 16,950 bp | Pathogenic |
| VCV000583426.1 | chr15: ?_25339117-25375783_? | NC_000015.9:g.(?_25584264)_  (25620930_?)dup | 36,667 bp | VUS |
| VCV000417431.1 | chr15: ?_25337249-25405506_? | NC_000015.9:g.(?_25582396)_  (25650653_?)dup | 68,258 bp | VUS |
| VCV000458664.6 | chr15: ?_25339117-25409127_? | NC_000015.9:g.(?_25584264)_  (25654274_?)dup | 70,011 bp | Pathogenic |
| this case | chr15:  25364087-25387427 | seq[GRCh38]  NC_000015.10:g. 25364087-25387427dup mat | 23,340 bp | Pathogenic |
